# Supportive care for cancer-related symptoms in pediatric oncology: a qualitative study among healthcare providers

**DOI:** 10.1186/s12906-023-03924-x

**Published:** 2023-04-03

**Authors:** Dana C. Mora, Miek C. Jong, Sara A. Quandt, Thomas A. Arcury, Agnete E. Kristoffersen, Trine Stub

**Affiliations:** 1 National Research Center in Complementary and Alternative Medicine (NAFKAM), Faculty of Health Science, Department of Community Medicine, UiT The Arctic University of Norway, Tromsø, N-9037 Norway; 2 Department of Epidemiology and Prevention, Division of Public Health Sciences, Wake Forest University School of Medicine, Winston-Salem, NC 27157 USA; 3 Department of Family and Community Medicine, Wake Forest University School of Medicine, Winston-Salem, NC 27157 USA

**Keywords:** CAM, Integrative medicine, Pediatric oncology, Qualitative, Resilience, Supportive care modalities

## Abstract

**Background:**

The aim of this study is to gain insight into the clinical experiences and perceptions that pediatric oncology experts, conventional healthcare providers, and complementary and alternative medicine (CAM) providers in Norway, Canada, Germany, the Netherlands, and the United States have with the use of supportive care, including CAM among children and adolescents with cancer.

**Methods:**

A qualitative study was conducted using semi-structured in-depth interviews (*n* = 22) with healthcare providers with clinical experience working with CAM and/or other supportive care among children and adolescents with cancer from five different countries. Participants were recruited through professional associations and personal networks. Systematic content analysis was used to delineate the main themes. The analysis resulted in three themes and six subthemes.

**Results:**

Most participants had over 10 years of professional practice. They mostly treated children and adolescents with leukemia who suffered from adverse effects of cancer treatment, such as nausea and poor appetite. Their priorities were to identify the parents' treatment goals and help the children with their daily complaints. Some modalities frequently used were acupuncture, massage, music, and play therapy. Parents received information about supplements and diets in line with their treatment philosophies. They received education from the providers to mitigate symptoms and improve the well-being of the child.

**Conclusions:**

Clinical experiences of pediatric oncology experts, conventional health care providers, and CAM providers give an understanding of how supportive care modalities, including CAM, are perceived in the field and how they can be implemented as adaptational tools to manage adverse effects and to improve the quality of life of children diagnosed with cancer and the families.

## Background

Cancer is the leading cause of death for children and adolescents around the world [1, 2]. Overall, estimated annual incidence rates vary between 50 and 200 per million in children under 15 years of age, and between 90–300 per million individuals who are in the age group of 15 to 19 years old [1]. The overall incidence of cancer among children (0–17) in Norway is 170 per million [3], which is similar to the rest of Europe [4]. The types of cancers that occur in children mainly comprise neoplasms of the blood and lymphatic system (leukemia or lymphoma), embryonal tumors (e.g., retinoblastoma, neuroblastoma, nephroblastoma), and tumors of the brain, bones, and connective tissues [5]. In high-income countries, including Norway, 80% of children survive their cancers, but there are significant variations depending on the tumor type [6]. In low- and middle-income countries, only about 20% survive [7].

Most children survive cancer with conventional medicines, and the treatment protocols vary according to diagnosis. For leukemia and lymphomas, the treatment is chemotherapy [6, 8]. Brain tumors are treated with surgery, chemotherapy, or radiotherapy. Other tumors are most often treated with surgery in addition to chemotherapy [8]. Children have a developing body, and cancer treatment may cause strong adverse effects. Radiotherapy, especially, can damage the healthy tissue of the brain, skeleton, and metabolic system, as well as other organs that are not fully developed [8]. When children receive treatment, it is common for the immune system to weaken. This means that the child is susceptible to infections, which, for a period of time, means that the child cannot participate in normal activities such as school, daycare, and group leisure activities. Moreover, children receiving treatment must live with any consequences of treatment for the rest of their lives [9].

The burden brought about by conventional medicine treatments has led parents to seek different complementary and alternative medicine (CAM) modalities within supportive care [10]. Supportive care is defined by the United States National Cancer Institute as care given to improve the quality of life of people who have an illness or disease by preventing or treating, as early as possible, the symptoms of the disease and the side effects caused by treatment of the disease. Supportive care includes physical, psychological, social, and spiritual support for patients and their families [11]. CAM is defined as a group of diverse medical healthcare system practices and products that are not considered part of conventional medicine [12]. Different countries have different definitions and regulations for CAM [13]. What is considered CAM in one country might not be considered CAM in another country. Hence the umbrella term of supportive care describes well the different modalities used in integrative care. Integrative health care is a caring approach that involves bringing together complementary and conventional treatment approaches in a coordinated manner to address an individual's health needs [14]. Although CAM modalities alone have not proven to be effective for cancer treatment, using them as complements to conventional medicine has been shown to improve the health of cancer patients [12]. Studies have reported that massage therapy [12] and acupuncture [15, 16] among others provide benefits to children during cancer treatment. A systematic review of randomized controlled trials (RCT) from 2022 [17] showed that CAM, including acupuncture and hypnosis, reduces chemotherapy-induced nausea and vomiting.

This research team carried out a focused ethnographic study through semi-structured interviews of families of children with cancer in Norway [18]. Results showed that parents are interested in discussions about CAM and other supportive care modalities that help them to care for themselves, their children, and their families (i.e., reduce anxiety, make healthy food, and keep a normal daily routine). Parents reported they prefer to obtain CAM information from reliable sources such as conventional healthcare providers (doctors or nurses).

Although oncologists generally discuss treatment options with patients, they largely ignore CAM [19]. A 2016 national survey among oncology experts and CAM providers in Norway found that the majority of medical doctors and nurses believed that it is risky to combine CAM and conventional cancer treatment (78% and 93%, respectively). Eighty-nine percent believed that CAM modalities should be subjected to more scientific testing before being accepted by conventional healthcare providers. This contrasts with 57% of the CAM providers [20]. Thus, the philosophical divergence of conventional and CAM approaches to health has often resulted in professional tension between conventional and CAM providers, resulting in opposition to CAM use and integration in parts of the medical community [21, 22]. This situation puts patients who use CAM at risk because they are resistant to disclosing their CAM use to their health care team.

Therefore, to gather more nuanced information about the use of CAM and other supportive care modalities in childhood cancer, we aimed to collect information from different healthcare providers with clinical experience in the area. We hoped that their experiences with supportive care modalities can provide another perspective and contribute to new insight in a field that is under-researched.

The aim of this study is to gain insight into the clinical experiences and perceptions that pediatric oncology experts, conventional healthcare providers and CAM providers in Norway, the United States, the Netherlands, Germany, and Canada have with the use of supportive care, including CAM, among children (0–9) and adolescents (10–19) [23] with cancer. To reach our aim we interviewed pediatric oncology experts (pediatric oncologist and nurses), other conventional healthcare providers (physiotherapists, nutritionists, and play therapists), and CAM providers (acupuncturists, healers, and massage therapists).

## Methods

This is a qualitative study [24], consisting of 22 semi-structured individual interviews. Qualitative design is useful when examining a phenomenon of previously limited knowledge [25]. It is important to understand the philosophical and medical context of supportive care modalities including CAM. A qualitative design is suitable for generating such information [26, 27].

### Study area and setting

This study was conducted in Norway, but healthcare providers from different countries (Canada, the Netherland, Norway, Germany, and the United States) were interviewed. Norway follows the Nordic health model of universal health care [28]. Canada [29], Germany [30], and the Netherlands [31] also have universal health care systems. The United States has multiple health systems that operate independently. The private sector plays a stronger role where private third-party payer sources (i.e., insurance companies) cover more than half of Americans' health expenses [32]. In all the countries, regardless of the healthcare system, supportive care modalities such as CAM are mostly offered outside the conventional healthcare system.

### Inclusion criteria

Inclusion criteria for healthcare providers were: (1) trained as pediatric oncology expert (doctor, nurses); conventional healthcare providers other than doctors and nurses; or CAM provider (practicing at least one or more CAM modalities inside or outside the conventional healthcare system) and (2) clinical experience working with supportive care and/or CAM modalities among children (0–9 years) and/or adolescents (10–19 years) [23]with cancer.

### Recruitment

Participants were recruited using purposive sampling [33] and were contacted through email and telephone. The researchers had no prior relationships with the individual participants. The Norwegian participants were recruited through the University Hospital of North Norway (UNN) (*n* = 5); the Norwegian Healer Association (*n* = 2); the Norwegian Homeopathy Association (*n* = 1); the Acupuncture Association (*n* = 1) and Norwegian Association for Psychotherapy (*n* = 1). The providers outside of Norway were recruited through the research team's professional networks in Canada (*n* = 1), Germany (*n* = 1), the Netherlands (*n* = 3), and the United States (*n* = 7).

### Participants

Before completing the interviews, the researchers informed the participants about the aim of the study and the purpose and content of the interview. Written and verbal informed consent was obtained from all participants. The study participants were informed that they could withdraw from the study for any reason at any time. The study was approved by the Norwegian Center for Research Data, reference number 978969. None of the participants dropped out.

### Data collection

Interviews were semi-structured, and an interview guide was developed by the investigators based on an review of the existing literature [34] and their knowledge of the field. Eight interviews were conducted face-to-face at workplaces (*n* = 7) and a private home (*n* = 1). Fourteen were conducted via Teams (a cloud-based video conferencing platform). The interviews were audio-recorded with the consent of the participants. Most of the interviews took between 30–60 min to complete. The first author (DCM) performed the interviews (*n* = 12) in English, while the last author (TS), who is Norwegian, performed the Norwegian interviews (*n* = 10) in Norwegian. To ensure the anonymity of each participant, they received an identification number (ID#). Field notes were taken during the interviews. The interviewers had previous experience conducting qualitative research [35–38], both interviewers are females and worked conducting research related to CAM at the time of the interviews.

### Data analysis

The interviews were transcribed verbatim into English by the first author (DCM). All the Norwegian interviews were transcribed verbatim by a professional service and translated into English by the senior author (TS). The analysis was conducted using conventional content analysis [39]. The success of content analysis depends on the coding process and in this study the codes were defined during the data analysis. The data were coded inductively, the codes were generated after DCM and TS carefully read the interviews. The data were entered and coded into Nvivo 1.61 [40]. After reviewing the coding both authors discussed any disagreements. The themes were developed by the first and the senior authors after reading and reviewing the interviews separately. Three themes were identified: (1) Perceptions of supportive care (2) Implementation of supportive care (3) the Empowerment of parents and overall care for the family. After identifying the three themes, six subthemes were developed (Table 1). Transcripts were not returned to participants for comment and/or correction. The consolidated criteria for reporting qualitative studies (COREQ) [41] were followed to ensure the methodological quality of the study. All methods were carried out in accordance with relevant guidelines and regulations.

**Table 1:** Overview of the main themes and subthemes

Themes	Subthemes
Perceptions of supportive care	- Clinical practice - Effect of supportive care - Supportive care for adverse effect management - Supportive care for palliative care
Implementation of supportive care	- Adverse effects management
Family empowerment and overall care for the family	-Providing agency, comfort, and relief

## Results

Twenty-two pediatric oncology experts, conventional health care providers, and CAM providers were recruited. Most participants were female with a mean age of 45 years (range 25–68 years). Over 70% of the participants (*n* = 17) had ten or more years of experience in clinical practice (Table 2).

**Table 2:** Demographic data of the participants

**Health care providers**	**Total (*n* = 22)**	**Oncology Experts (*n* = 11)**	**Conventional**^a^ **(*n* = 4)**	**CAM providers**^a^ **(*n* = 7)**
	**n (%)**	**n (%)**	**n (%)**	**n (%)**
**Gender**				
Female	18 (82)	8 (73)	4 (100)	6 (86)
Male	4 (18)	3 (27)	0 (0)	1 (14)
**Age** (mean)	45.5	48.3	46.5	51.6
18 – 40 years of age	6 (27)	3 (27)	2(50)	1 (14)
41—60 years of age	10 (45)	6 (55)	1 (25)	4 (57)
61 years and older	6 (27)	2 (18)	1 (25)	2 (29)
**Years in practice**				
0–10 years	5 (23)	2 (18)	2(50)	1 (14)
11–20 years	8 (36)	3 (27)	0 (0)	4 (57)
21–30 years	4 (18)	3 (27)	1(25)	1 (14)
More than 31 years	5 (23)	3 (27)	1 (25)	1 (14)
**Training**^a^				
Acupuncturist^a^	5(18)	3 (27)	0 (0)	2 (14)
Anthroposophic medicine^a^	1 (5)	1 (9)	-	-
Healer	3 (14)	1 (9)	-	2 (14)
Homeopath	1 (5)	-	-	1 (7)
Nurse^a^	5 (23)	3 (27)	-	2 (14)
Massage therapist	1 (5)	-	-	1 (7)
Music therapist	1 (5)	-	-	1 (7)
Nutritionist	2 (9)	-	2 (50)	-
Pediatric oncologist^a^	6 (27)	3 (27)	-	3 (21)
Physiotherapist^a^	1 (5)	-	1 (25)	-
Play therapist	1 (5)	-	1 (25)	-
Psychodrama therapist^a^	1 (5)	-	-	1 (7)
**Sector**				
Public sector	9 (45)	6 (55)	2 (50)	1 (14)
Private sector	9 (36)	5 (45)	2 (50)	2 (29)
Self-employed:	4 (18)	-	-	4 (57)
**Country**				
Canada	1 (5)	1 (7)	-	-
Germany	1 (5)	1 (7)	-	-
The Netherlands	3 (14)	2 (13)	-	1 (14)
Norway	10 (45)	4 (36)	2 (50)	4 (57)
United States	7 (32)	3 (27)	2 (50)	2 (29)

^a^These providers were trained as both conventional and CAM providers

Fifteen of the participants were conventional pediatric oncology providers or other conventional providers (6 were pediatric oncologists, 5 were nurses, 4 were other conventional health care providers (i.e., physiotherapists (1), nutritionists (2), play therapist—in Norway, play therapists are licensed conventional healthcare providers (1)).

Almost one-third (*n* = 4) were self-employed (healers, homeopath, massage therapist), and nine (*n* = 9) were employed in the public health care sector (nurse, physiotherapist, pediatrician, music and play therapist). One participant worked both inside and outside the official sector (physiotherapist and psychodrama therapist). Nine participants worked for private hospitals.

All the participants had experience working with pediatric oncology patients (aged 0–19 years old), and 18 worked in pediatric oncology settings. Five participants had training in both conventional care and CAM. Participants were recruited from five countries (Canada *n* = 1, Germany *n* = 1, Netherlands *n* = 3, Norway *n* = 10, United States *n* = 7) (Table 2).

### Perceptions of supportive care

Through this theme, insight into the clinical practices of participants is gained, as well as what perceptions oncology experts and conventional providers have of supportive care. Four subthemes emerged: clinical practice, supportive care for palliative care, effect of supportive care, and supportive care for adverse effect management.

#### Clinical practice

Most of the participants (ID 1, 2, 5–9, 11–13, 15) stated that the cancer diagnosis they treated most often was leukemia (acute lymphocytic leukemia (ALL) or acute myeloid leukemia (AML)). In Norway, patients with cancer are diagnosed and treated at one of the main four hospitals in the country: Oslo University Hospital, Haukeland University Hospital, St. Olav's Hospital, and University Hospital of North Norway. According to one participant (ID 11), patients most often have chemotherapy or surgery. If the child has a rare tumor or needs special surgery, they are referred to the main hospital, in Oslo or they might be sent to other countries for treatment. Outside Norway, participants also stated that most children are treated with chemotherapy, radiation, or surgery (ID 1,5, 6, 8–10, 22, 23). The symptoms from cancer treatment most often reported in the interviews were nausea, mental health issues such as anxiety, lack of socialization, and depression. In addition, pain, vomiting, fatigue, neuropathy, mucositis, constipation, decrease appetite, and insomnia are also common. Even though the medical systems varied from country to country, all the participants (ID 1–18, 22, 23) who worked in hospitals said that the supportive care modalities (e.g., play therapy, acupuncture, and music therapy) offered at the hospital are free for the patient, but parents must pay out-of-pocket for any modalities performed outside of the hospital (e.g., acupuncture, healing, and massage).

All the conventional care providers interviewed outside of Norway had experience working in integrative medicine settings and had positive beliefs about CAM to various degrees. One oncologist (ID 22) stated that “a lot of CAM treatments would be okay to use but there is just not enough research”. However, another pediatric oncologist (ID 9) was more skeptical about the modalities, he stated, “I'm not very much in favor, let's be clear, I'm not in favor of prescribing these things [modalities], which cost a lot and are not proven.”

A program manager and CAM provider in the United States (ID 2) stated that, in her program, they view supportive patient care through three different lenses. These lenses are prevention, mitigation of adverse effects, and long-term effects. The treatment plan for the different supportive care modalities is discussed among the provider, the parents, and the child, depending on the diagnosis, conventional treatment, and most importantly the immediate (daily) needs of the child. The conventional care team is not usually involved unless there is a specific question or someone in the conventional care team is trained as a CAM provider. In one program, consultations with the CAM provider often happen soon after diagnosis to focus on prevention and mitigation of symptoms from conventional cancer treatment. An acupuncturist (ID 3) explained that her job at the time of consultation, given all the other treatment the child was enduring, was to “have a flexible toolbox and prevent things from happening but also mitigate what is going on in the moment and just support [the patient] in the moment. …the overriding goal is just to help in the moment if possible.”

Even in integrative programs, integrative medicine is not offered/discussed with all of the patients. In most programs, supportive care modalities including CAM (such as acupuncture, massage, or reiki) are only offered if the parents or patient asks for it or if someone in the oncology treatment team recommends integrative care for that patient.

#### Effect of supportive care

Providers also believe that it is okay to use CAM and are willing to recommend it as long as it does not add extra burden for patients. A provider (ID 15) stated “There must be evidence of effect of CAM. I think that the treatment must not cause additional suffering for the child just so the parents can feel that they have tried it…If the treatment has effect and does not harm the child, I could recommend it.” Other providers (ID 4, 22) who recommend CAM to manage adverse effects from cancer treatment believe that some modalities are flagged on the conservative end but that many modalities would be fine to use, there is just evidence lacking. A providers (ID 4) stated “… there is evidence supporting the use [of CAM] in patients in outpatient setting, but there is very little data.”

#### Supportive care for adverse effect management

For all the participants supportive care is used to help children manage adverse effects from cancer treatment not to treat cancer itself. As an oncologist (ID 1) stated, “So, we don't ever treat the cancer directly. We treat the adverse effects of the cancer, and we try to approach the patient at diagnosis and at initiation of treatment.”

Two pediatric oncologists (ID 5, 22) also stated that they recommend supportive care as a non-pharmacological treatment to manage symptoms. An oncologist (ID 5) expressed that the last thing patients want to do to manage symptoms is to take another pill. She stated that “there are symptoms like fatigue, anxiety, insomnia, that we just don't have the interventions for. I am thinking there has got to be a better way to make people feel better as they're going through their cancer treatments that doesn't just involve asking, particularly children, to take more medicines”.

#### Supportive care for palliative care

Conventional healthcare providers and oncology experts interviewed are more open to supportive care for those in palliative care. A nurse (ID 12) stated, “When the story ends, the parents should be left feeling that they did whatever they could for their child. It has never been a problem to get a healer to come here [at the hospital], upon request from the parents”. Likewise, a pediatric oncologist (ID 22), while discussing the use of supplements, stated that she recommends certain treatments depending on the prognosis. For example, for ALL she does not recommend taking extra substances [herbs or supplements] due to concerns of decreasing the chemotherapy efficacy or increasing the toxicity. However, if the child is at the end of life she stated, “I'm much more liberal with that [using supplements]. I would be like yes, if that's not going to hurt you, fine.”

In Norway specifically, most of the conventional care nurses are skeptical about supportive care modalities, especially CAM. They all had limited knowledge of CAM and agreed it should be used as a last resource when nothing else has worked to enable parents to give the best care for their child.

### Implementation of supportive care

Throughout this theme, the participants describe various modalities they used and how they helped the child cope with adverse effects from conventional cancer treatments.

#### Adverse effects management

Most of the modalities mentioned by the participants are recommended and used to manage the adverse effects of cancer treatment. Among the modalities mentioned in the interviews were acupuncture, healing, massage/aromatherapy, nutrition (herbs, dietary changes, and supplements), and mental health (art, music, play therapy, and psychodrama).

##### Acupuncture

According to the participants (ID1, 2, 3, 4, 5, 10, 22), acupuncture is one of the modalities often used and recommended in the United States. In pediatric oncology, the modality is mostly used to reduce symptoms from conventional cancer treatment, and it is considered safe. In one program, acupuncture is offered to the patients depending on the chemotherapy regimen the patient is receiving and the potential adverse effects that might be derived from that treatment (ID 2, 3). All acupuncturists use needles, acupressure, ear seeds, laser, or acupuncture bands. A Norwegian acupuncturist working in private practice stated that because children have simple patterns of imbalance, not many needles are needed. When treating children, thin short needles are used as they are gentler. Acupuncturists, pediatric oncologists, and nurses said that they use acupressure points to relieve nausea in their patients. For example, a pediatric oncologist (ID 22) says that she recommends acupressure for children who have refractory nausea and vomiting. An acupuncturist (ID 18) who works in an integrative program stated, “you should not treat children as adults” and noted that an individual assessment should always be made. Most providers agreed that babies, younger children, and teenagers tolerate needles, so they are often used. According to the participants, children between 5–12 years are more afraid of needles, and acupressure or laser are more often used with that age group as they are not as invasive. Acupuncture is also recommended for pain, functional limitation due to neuropathy, musculoskeletal limitation, anxiety, relaxation, and constipation.

##### Nutrition

Some of the symptoms that are addressed through nutrition are vomiting and nausea. Providers use herbal teas such as peppermint or ginger and add fresh ginger to smoothies to aid with nausea and vomiting symptoms. For those with mouth sores, providers recommend soft and bland foods and avoiding hot spicy foods. As described by the nutritionist (ID 6) below:

“Kids do better when they are able to sip on something through a straw throughout the day than having to actually eat.”

Children who lack appetite can try small protein pack snacks throughout the day (proteins can include dairy, meat, nut butter, and legumes). Commonly available sources of proteins for children can be milk, yogurt, and cheese. They can also try smoothies that are calorie and protein dense. For those who, due to chemotherapy, are sensitive to smells, the nutritionist recommends eating foods that are cold or at room temperature. Nutritionists (ID 6, 7) counsel parents based on food preferences, family eating patterns, accessibility to different foods, and cultural food practices.

According to nutritionists (ID 6, 7), avoiding foods with concentrated sugars or carbohydrates and having a source of healthy fat (e.g., olive oil, avocado oil, fatty fish, seeds, nuts) or protein and complex carbohydrates such as oatmeal or whole grains can help children with fatigue. The nutritionists also talk to parents about tube-feeding formulas. After addressing the basics when selecting a formula (does the child tolerate it? do they need elemental -broken down, hydrolyzed for easier digestion- or intact?), the nutritionist tries to involve the parents as much as possible to select the formula that is tolerated best by the child. They involve the parents by reviewing the ingredients and reviewing previous experiences based on knowledge from other parents and patients.

##### Healing/Reiki

Participants referred to healing, reiki, and healing touch in the interviews. According to the Norwegian Law of Alternative Treatment [42], healers and other CAM providers are not allowed to treat cancer itself, but the healing may be used to strengthen the body and to treat the adverse effects of cancer and treatment. This is illustrated in the quotation below:

*“*Parents are interested in healing that strengthens the immune system and provides children with enough energy to face what they must go through (ID 12).”

A participant (ID 20), who works as a healer, prepares herself before treating the child by processing her emotions, meditating, and asking for the power to help perform the healing of the child. Most of the children treated by the healer are diagnosed with leukemia and brain tumors. For the healers (ID 12, 20), included in this study, the primary focus is to provide trust, strengthen the child's energy and aura, and relieve pain. The treatments are only given during the children's breaks from chemotherapy or radiation. Healers do not treat the area in which the tumor lies, but the areas around it. Sometimes, the participant (ID 20), treats both the parents and the child.

##### Massage/Aromatherapy

Providers also recommend modalities such as massage and play therapy for general well-being and to make the stay at the hospital or home with a sick child as normal as possible. Modalities often used for this purpose are massage and aromatherapy. According to an oncologist (ID 1), chemotherapy and radiation therapy cause muscle tension and dryness of the muscles and the joints; massage is recommended for loosening the muscles and tendons in the body. According to a massage therapist, massage is used to help the child relax and loosen the body, and to decrease anxiety, stress, and fatigue. Another factor that is taken into consideration when performing massage is the physiological and emotional impact of a cancer diagnosis on the family. Different providers (ID 3, 8, 10, 13) stated that they teach the parents message so that they can help their children. In addition, it is used for sleep problems, to reduce head and neck pain, and musculoskeletal complaints. A massage therapist (ID 23) stated that she works under the principle that *less is more*. During treatment, the massage sessions last 20—30 min maximum and can only be done on part of the body. However, the first session often lasts only 10 min to make sure it is safe, depending on the patient and their health history. Apart from using needles, acupuncturists use tui na massage (tui na follows the assumptions of Chinese medicine, it is a system of massage, manual acupuncture point stimulation, and manipulation) [43]. One acupuncturist (ID 3) used tui na to help constipated children. One of the chemotherapy drugs (Vincristine®) causes constipation. A new dose cannot be administered until children have a bowel movement, so the acupuncturist uses tui na and acupressure to help calm the nervous system and move the bowel. In many cases, this massage has reportedly been effective.

Aromatherapy is also offered in integrative programs in the United States and Germany because, like massage, it has been shown to mitigate chemotherapy's effects and be safe. According to the participants, it is more often used for improving nausea, vomiting, sleep, and anxiety. The programs that offer this modality have trained personnel who prescribe the oils and make personalized nasal inhalers for the children. The oils are sometimes used with massage or acupressure for relaxation and constipation (ID 3). Ginger, lemon balm, and peppermint teas are incorporated together with deep breathing to help the children manage nausea caused by chemotherapy in one of the programs (ID 7). Lavender extract is also used to massage children's feet and lower extremities to help children sleep (ID 8).

##### Play, psychodrama, and music therapy

Diverse modalities like play, psychodrama therapy, music, and virtual reality programs are often used for stress management, to divert the attention of children from painful procedures, treatment regimens, and the burden of having a cancer diagnosis. At the hospital, children play to process emotions, and through role-play, they cope with their situation. Using techniques such as role-play, the provider helps children process their emotions. The playroom is a safe space where doctors and nurses are not allowed and where both patients and parents can unfold their emotions. In Norway, the play therapist can also collaborate with other providers (e.g., the physiotherapist) to help children practice motor skills and language development.

Psychodrama is another strategy offered to help pediatric oncology patients express their emotions. Psychodrama is implemented by following three pillars: mirroring, role-playing, and duplication. Children use play to mirror their emotions. As stated by the therapist (ID 17), “Whatever the children have experienced will be symbolically expressed in the play.” For example, the feeling of being powerless is often expressed in play when the child gets sleepy, disappears, or becomes dizzy. Children can go quickly in and out of roles; through role-play, the child can regain mental and physical control. For instance, a child with cancer expressed her feelings of powerlessness during therapy. In the session, she played a guard that captured a prisoner [the therapist], provided lousy food to the prisoner, and told her she would be in prison forever. The child wanted the therapist to feel/experience the same feelings as she did during cancer treatment, and through that, the child processed her own feelings.

Music therapy is used for distraction, relaxation, and as a means of visualization. A provider (ID 16), for example, can listen to music with the patient and while the music is playing the patient is guided to relaxation. Music therapy is also used for parents, by playing music parents can express their emotions, including the realization that they are scared by their child's diagnosis, but, at the same time, they need to be the safety net to comfort the child. This is a dilemma for the parents. They need to be strong, but they are also afraid, something they try to hide from the child. As stated by the therapist during this time, “It is important to strengthen relations in the family.” The provider works with different instruments, including piano, guitar, and flute sound sticks. Music is used to strengthen family relationships and allow the children to express their emotions. For example, the provider had a little girl who stopped talking after surgery. During a music therapy section a week after surgery, the music therapist and the girl were looking for the girl's voice. They found the voice inside the guitar by playing lullabies. Having found her voice, the girl started to talk again. The music therapist uses puppet dolls to help the children express their feelings. She has a crow who is moody, sad, and angry; she also has a kitten who is anxious and worried. The puppets give the child different conversation partners that help them open up and talk to the puppets about anything of interest.

### Empowerment of parents

Lastly, a theme emerged that captured the providers' perceptions of the parent's role during the treatment of the child and how supportive care provides a way for parents to feel they are actively part of their child's care.

#### Providing agency, comfort, and relief

The high survival rates of childhood cancer are due to closely prescribed treatment protocols. These protocols are strictly implemented. The pediatric oncologist takes complete control, and the parents have limited agency in making decisions about their child's treatment, potentially creating a feeling of helplessness among the parents. It is the providers' impression that the parents often feel afraid because of their child's diagnosis, but at the same time, they feel the responsibility to provide safety and comfort and want to do everything in their power to help the child. As described by a pediatric oncologist (ID 8):

*“*Pediatric oncology is very passive [for the parents], parents sign the informed consent, and then we [pediatric oncologists] give to the children any drug or intervention. So, the parents, at some stage, just have to tolerate it.”

All participants expressed that parents experience a passive role and a loss of authority and control that can lead them to anxiety and worry. Given the lack of agency parents have during conventional cancer treatment of their children, all the providers agree that the use of supportive care, including CAM helps parents overcome some anxiety and gives them back control. One acupuncturist (ID 3) stated: “CAM gives a sense of control, a sense of contribution, which can be therapeutic. By educating them about all the ways that exist and can be used to mitigate or treat symptoms, parents are given back agency*.”*

As discussed in the former results, supportive care modalities give parents the agency to establish a treatment plan together with the CAM provider. For example, by learning about acupressure, they can use specific points to manage nausea and vomiting at home.

Education is an important tool used by providers to give the parents agency, provide some comfort to the children, and provide a sense of normalcy to the family. Often using things daily that are helpful, and teaching and empowering parents and children to do some of those things (e.g., massage, acupressure) has a significant impact because providers can see those patients and their parents feel better. A pediatric oncologist (ID 1) stated:

“Parents feel involved because they can do these things. That is a huge win and that is an everyday thing. So, to me, those everyday things are bigger than any other big miraculous thing.”

Providing treatments such as acupuncture or massage to parents is another technique providers use to help parents cope with their child's cancer diagnosis and treatment. In the providers' perception offering these treatments to parents helps mitigate some of the fears or questions both the parents and patients have about supportive care modalities.

## Discussion

The participants interviewed are a heterogeneous group with different years of experience, different professions, and from different countries; however, common themes emerged from their interviews. They spoke about improving the general well-being of the patients and their families by empowering them to take control of the cancer treatment using supportive care modalities. For example, parents are taught how to give massages to help their children go to sleep or help with constipation. They also shared details about their perceptions of supportive care including their clinical practice, such as how their programs are coordinated and what and how supportive care modalities are offered and implemented. Participants also reported having similar experiences and goals concerning the treatment of children with cancer and the use of supportive care. For instance, most providers recommended supportive care to manage symptoms from cancer treatment such as nausea, anxiety, and depression. The supportive care modalities most often mentioned to help mitigate these adverse effects were massage, nutrition, play therapy, and acupuncture.

Well-established programs in pediatric oncology that integrate CAM modalities and conventional treatments exist in different parts of the world, including Europe and North America. Programs at university hospitals in the United States [44] and Germany [45] offer acupuncture, anthroposophic medicine, aromatherapy, exercise and movement therapy, herbal and homeopathic remedies, massage, mind–body medicine, and art therapy. While they are becoming more common [46], integrative programs in pediatric oncology are limited [47]. A survey from Jacobsen et al., [48] reported that CAM was offered in 64.4% of the hospitals in Norway in 2013. In Norway, CAM is normally not offered in pediatric oncology settings. However, other supportive care modalities such as music therapy, art therapy, and play therapy are offered to varying degrees in all four main hospitals. No major differences were found between public and private, nor between non-psychiatric and psychiatric hospitals. Acupuncture (37.3%) was the most commonly offered modality followed by art and expression therapy (25.4%), massage (15.3%), and alternative diet (8.5%). On the other hand, music therapy was offered by 13.6% of the hospitals [48]. Music therapy is a popular modality among children and, according to the participants in this study, is commonly offered at pediatric oncology units in Norway. Art therapy, play therapy, and clowns are other supportive care modalities offered in children's wards (including oncology) in Norway [49–52]. Even though CAM is used by pediatric oncology patients [53], according to the literature, there is a lack of knowledge about CAM among pediatric oncologists [54–56].

The results of our study showed that although supportive care modalities are used, they are not routinely offered to all pediatric oncology patients. All the participants in our study reported open communication about supportive care, including CAM; however, children are referred to integrative programs only if parents ask about CAM. This mirrors a skeptical attitude toward these modalities among many healthcare providers, which is in line with the existing literature regarding the attitudes of conventional health providers about CAM. In a study about attitudes of pediatric oncologists, it is reported that only 41% of the oncologists raise the topic of CAM during the first consultation [55]. The same study [55] also reports that over 70% of the pediatric oncologists agree somewhat or fully that CAM should be used when all conventional therapies fail, also supporting responses obtained through our interviews. The latter is consistent with perceptions reported in this study, where providers are more favorable of supportive care, including CAM, during palliative care.

According to the participants in this study, supportive care modalities are an important component of care that can guide future clinical practice. The goal of applying supportive care is to improve the quality of life of children with cancer and their families by treating the adverse effects caused by cancer treatment. Modalities such as acupuncture [15, 16, 57–59], massage [12], aromatherapy [60], healing [61], music [62], play therapy [63], and psychodrama [64] have beneficial outcomes in children [17, 65]. In general, we found that supportive care modalities are used to provide comfort and control to the patients and parents; this is in line with other studies [66–68].

Due to the strict childhood cancer treatment protocols, parents report very little control over the uncomfortable and painful procedures and treatments the child has to endure after receiving a cancer diagnosis [68]. An important topic that emerged from these interviews is the empowerment that the use of supportive care provides to children and adolescents with cancer and their parents. Using different supportive care modalities to treat symptoms and complaints at home helps the families get back to normal everyday life even though the child is ill. This sentiment is in line with what Masten [69], called *the power of the ordinary*. This sentiment states that “resilience comes from the everyday magic of the ordinary, it comes from normative human resources in the minds, brains, and bodies, of children, in their families and relationships, and their communities.” [69] By creating daily routines with massage, taking control of the child's diet, or creating spaces where children can play, or listening to music, a sense of normalcy is created. This need for normalcy and family routines in times of adversity is in line with goals of parents found in a Norwegian study among parents who have children with cancer [35].

### Strengths and limitations

The findings of this study should be interpreted considering its limitations. The study centered on a small group of oncology pediatric experts, conventional health care, and CAM providers who were interviewed once, and all the participants interviewed were from high-income countries. An error introduced when the study population does not represent the target population is understood as selection bias [70]. Ideally, the subjects in a study should be very similar to one another and to the larger population from which they are drawn. If there are important differences, the results of the study must be understood with caution, which is the case in this study. Different modalities of CAM are offered/used by children and adolescents with cancer [53] and it was not possible to interview a provider for each modality. If more healthcare providers had been interviewed, or if multiple interviews had been done with each participant, it could have been possible to gather additional information about their clinical practice and their experience; however, no new information was achieved after 20 interviews, demonstrating that saturation was reached [71].

To our knowledge, this is the first study that interviews pediatric oncology experts, conventional healthcare providers, and CAM providers employing supportive care modalities among children and adolescents with cancer. The results show similarities in perceptions of supportive care use, the implementation of supportive care, and their approach to empowering parents during cancer treatment. This is important because it offers further knowledge and understanding of how conventional medicine and CAM clinical practices are used in combination to improve well-being, give hope, and treat adverse effects of cancer treatment among these children.

### Implications for practice

Understanding the implications that supportive care can have for children and their parents can help guide treatment protocols for children with cancer across different countries. Although countries have different healthcare systems, childhood cancer is a rare disease. In most high-income countries, the survival of childhood cancer has improved due to the integration of clinical research into front-line care from multidisciplinary specialists [72]. The ailments and needs of the children undergoing cancer treatment are similar across countries, particularly among children in high-income countries. Hence, the results of this research can offer modalities that focus on the overall well-being of the patients and their families. The information gained in this study can be used to inform other countries where supportive care is not integrated on how existing programs work, how they are integrated, and what modalities are used among this patient group. The results can also be used as evidence to generate practical guidelines, for example, in nursing to implement modalities such as massage and reiki. The finding regarding the empowerment of the parents can be used as a baseline to further investigate among parents how supportive care empowers and helps them during and after diagnosis and treatment.

## Conclusion

The overall results of this study give providers, parents, and patients insight into how healthcare providers working in pediatric oncology perceive the role of supportive care modalities in this field. According to the participants, these modalities can be used to help manage adverse effects of cancer treatment, but they also act as an adaptational system to develop resilience and empower children and their families while undergoing cancer diagnoses and treatment. Through the development of resilience and empowerment, children can have better overall health outcomes that could lead to healthier, happier, and more productive lives during and after cancer treatment.

## Data Availability

All data generated or analyzed during this study are included in this published article.

## Abbreviations

CAM: Complementary and alternative medicine
RCT: Randomized controlled trials
ALL: Acute lymphocytic leukemia
AML: Acute myeloid leukemia

## References

[1] Steliarova-Foucher E, Colombet M, Ries LAG, Moreno F, Dolya A, Bray F, Hesseling P, Shin HY, Stiller CA, Bouzbid S (2017). International incidence of childhood cancer, 2001–10: a population-based registry study. Lancet Oncol.

[2] Bhakta N, Force LM, Allemani C, Atun R, Bray F, Coleman MP, Steliarova-Foucher E, Frazier AL, Robison LL, Rodriguez-Galindo C (2019). Childhood cancer burden: a review of global estimates. Lancet Oncol.

[3] National Quality Register for Childhood Cancer, Annual Report 2020. The Cancer Registry [https://www.kreftregisteret.no/globalassets/publikasjoner-og-rapporter/arsrapporter/publisert-2021/arsrapport-nasjonalt-kvalitetsregister-for-barnekreft-2020.pdf] Accessed.

[4] De Angelis R, Sant M, Coleman MP, Francisci S, Baili P, Pierannunzio D, Trama A, Visser O, Brenner H, Ardanaz E (2014). Cancer survival in Europe 1999–2007 by country and age: results of EUROCARE-5—a population-based study. Lancet Oncol.

[5] Jemal A, Vineis P, Bray B, Torre L, Forman D (2015). The Cancer Atlas New-York: Cancer in Children.

[6] Barnekreft. English: Pediatric cancer [https://www.barnekreftforeningen.no/barnekreft] Accessed 6 Oct 2019

[7] Howard SC, Zaidi A, Cao X, Weil O, Bey P, Patte C, Samudio A, Haddad L, Lam CG, Moreira C (2018). The My Child Matters programme: effect of public–private partnerships on paediatric cancer care in low-income and middle-income countries. Lancet Oncol.

[8] Lissauer T, Clayden G (2016). Pædiatri. English: Pedriatrics.

[9] Seneffekter etter behandling av kreft. English: Late effekts after cancer treatment [https://www.barnekreftforeningen.no/barnekreft/seneffekter] Accessed 4 May 2020

[10] Fitch M (2008). Supportive care framework. Can Oncol Nurs J.

[11] Dictionary of Cancer Terms : Supportive Care [https://www.cancer.gov/publications/dictionaries/cancer-terms/def/supportive-care] Accessed 22 July 2022

[12] Radossi AL, Taromina K, Marjerrison S, Diorio CJ, Similio R, Njuguna F, Afungchwi GM, Ladas EJ (2018). A systematic review of integrative clinical trials for supportive care in pediatric oncology: a report from the International Society of Pediatric Oncology, T&CM collaborative. Support Care Cancer.

[13] Wiesener S, Falkenberg T, Hegyi G, Hök J, Roberti di Sarsina P, Fønnebø V (2012). Legal Status and Regulation of Complementary and Alternative Medicine in Europe. Complement Med Res.

[14] Kania-Richmond A, Metcalfe A (2017). Integrative health care - What are the relevant health outcomes from a practice perspective? A survey. BMC Complement Altern Med.

[15] Reindl TK, Geilen W, Hartmann R, Wiebelitz KR, Kan G, Wilhelm L, Lugauer S, Behrens C, Weiberlenn T, Hasan C (2006). Acupuncture against chemotherapy-induced nausea and vomiting in pediatric oncology Interim results of a multicenter crossover study. Support Care Cancer.

[16] Gottschling S, Reindl TK, Meyer S, Berrang J, Henze G, Graeber S, Ong MF, Graf N (2008). Acupuncture to alleviate chemotherapy-induced nausea and vomiting in pediatric oncology - a randomized multicenter crossover pilot trial. Klin Padiatr.

[17] Mora DC, Overvåg G, Jong MC, Kristoffersen AE, Stavleu DC, Liu J, Stub T (2022). Complementary and alternative medicine modalities used to treat adverse effects of anti-cancer treatment among children and young adults: a systematic review and meta-analysis of randomized controlled trials. BMC Complement Med Ther.

[18] Stub T, Arcury TA, Kristoffersen AE, Jong MC, Quandt SA: Parents of children with cancer: Strategies for managing care of sick child in the context of family life. Unpublished data 2019.

[19] Brown PR, Alaszewski A, Swift T, Nordin A (2011). Actions speak louder than words: the embodiment of trust by healthcare professionals in gynae-oncology. Sociol Health Illn.

[20] Stub T, Quandt SA, Arcury TA, Sandberg JC, Kristoffersen AE (2018). Attitudes and knowledge about direct and indirect risks among conventional and complementary health care providers in cancer care. BMC Complement Altern Med.

[21] Dwyer JM (2011). Is it ethical for medical practitioners to prescribe alternative and complementary treatments that may lack an evidence base? - No. Med J Aust.

[22] Joyce P, Wardle J, Zaslawski C (2016). Medical student attitudes towards complementary and alternative medicine (CAM) in medical education: a critical review. J Complement Integr Med.

[23] Adolescent health [https://www.who.int/health-topics/adolescent-health#tab=tab_1] Accessed 28 Feb 2023

[24] Quinn Patton M (2015). Qualitative research & evaluation methods. Integrating theory and practice.

[25] Kvale S, Brinkmann S, Anderssen TM, Rygge JF (2009). Det kvalitative forskningsintervju. English: The qualitative research interview.

[26] Fønnebø V, Grimsgaard S, Walach H, Ritenbaugh C, Norheim AJ, MacPherson H, Lewith G, Launsø L, Koithan M, Falkenberg T (2007). Researching complementary and alternative treatments - the gatekeepers are not at home. BMC Med Res Methodol.

[27] Malterud K (2001). Qualitative research: standards, challenges, and guidelines. Lancet.

[28] Magnussen J, Vrangbaek K, Saltman R (2009). EBOOK: Nordic Health Care Systems: Recent Reforms and Current Policy Challenges: McGraw-Hill Education (UK).

[29] Marchildon GP, Allin S, Merkur S (2020). Canada: Health system review. Health Syst Transit.

[30] OECD/European Observatory on Health Systems and Policies. Germany: Country Health Profile 2019. Paris: State of Health in the EU, OECD Publishing; 2019. 10.1787/36e21650-en.

[31] OECD/European Observatory on Health Systems and Policies. Netherlands: Country Health Profile 2019. Paris/Brussels: State of Health in the EU, OECD Publishing/European Observatory on Health Systems and Policies; 2019. 10.1787/9ac45ee0-en.

[32] Rice T, Rosenau P, Unruh LY, Barnes AJ, Saltman RB, van Ginneken E, World Health Organization. Regional Office for E, European Observatory on Health S, Policies (2013). United States of America: health system review.

[33] Patton MQ (2014). Qualitative research & evaluation methods: Integrating theory and practice.

[34] Stub T, Kristoffersen AE, Overvåg G, Jong MC (2020). An integrative review on the information and communication needs of parents of children with cancer regarding the use of complementary and alternative medicine. BMC Complement Med Ther.

[35] Stub T, Quandt SA, Kristoffersen AE, Jong MC, Arcury TA (2021). Communication and information needs about complementary and alternative medicine: a qualitative study of parents of children with cancer. BMC Complement Med Ther.

[36] Stub T, Kiil MA, Lie B, Kristoffersen AE, Weiss T, Hervik JB, Musial F (2020). Combining psychotherapy with craniosacral therapy for severe traumatized patients: A qualitative study from an outpatient clinic in Norway. Complement Ther Med.

[37] Quandt SA, Sandberg JC, Graham A, Mora DC, Stub T, Arcury TA (2017). Mexican Sobadores in North Carolina: Manual Therapy in a New Settlement Context. J Immigr Minor Health.

[38] Mora DC, Arcury TA, Quandt SA (2016). Good job, bad job: Occupational perceptions among Latino poultry workers. Am J Ind Med.

[39] Hsieh HF, Shannon SE (2005). Three approaches to qualitative content analysis. Qual Health Res.

[40] Richards L (2005). Handling Qualitative Data: A Practical Guide.

[41] Tong A, Sainsbury P, Craig J (2007). Consolidated criteria for reporting qualitative research (COREQ): a 32-item checklist for interviews and focus groups. Int J Qual Health Care.

[42] LOV-2003-06-27-64 Lov om alternativ behandling av sykdom mv. Act No. 64 2003 [Act relating to the alternative treatment of disease, illness, etc,] Helse og omsorgsdepartementet (Ministry of Health and Care Services). 2003. https://app.uio.no/ub/ujur/oversatte-lover/data/lov-20030627-064-eng.pdf. Accessed 15 July 2022.

[43] Ergil KV, Micozzi MS, Mayor D, Micozzi MS (2011). 5 - Qi in China's traditional medicine: the example of tuina. Energy Medicine East and West.

[44] Integrative Therapies [https://www.pediatrics.columbia.edu/about-us/divisions/hematology-oncology-and-stem-cell-transplantation/center-comprehensive-wellness/patient-care/integrative-therapies] Accessed 15 July

[45] Department of Pediatric Oncology and Pediatric Hematology [https://www.gemeinschaftskrankenhaus.de/medizin-therapie-pflege/fachabteilungen/kinder-/jugendmedizin/kinderonkologie/uebersicht/] Accessed 15 July 2022

[46] Rutert B, Stritter W, Eggert A, Auge U, Laengler A, Seifert G, Holmberg C (2021). Development of an Integrative Care Program in a Pediatric Oncology Unit. Complement Med Res.

[47] Längler A, Zuzak TJ (2013). Complementary and alternative medicine in paediatrics in daily practice—A European perspective. Complement Ther Med.

[48] Jacobsen R, Fønnebø VM, Foss N, Kristoffersen AE (2015). Use of complementary and alternative medicine within Norwegian hospitals. BMC Complement Altern Med.

[49] Barnekreft (Childhood cancer) [https://unn.no/behandlinger/barnekreft#vardesenteret] Accessed 15 July 2022

[50] Children's Department for Cancer and Blood Diseases [https://oslo-universitetssykehus.no/behandlinger/barnekreft?sted=barneavdeling-for-kreft-og-blodsykdommer#barne--og-ungdomsaktiviteter] Accessed 15 July 2022

[51] Will invest in music therapy [https://stolav.no/korus/vil-satse-pa-musikkterapi] Accessed 15 July 2022

[52] Children and young people in the hospital [https://helse-bergen.no/avdelinger/barne-og-ungdomsklinikken/barn-og-unge-pa-sjukehus#aktivitetar-for-barn-og-unge] Accessed 15 July 2022

[53] Bishop FL, Prescott P, Chan YK, Saville J, von Elm E, Lewith GT (2010). Prevalence of Complementary Medicine Use in Pediatric Cancer: A Systematic Review. Pediatrics.

[54] Alnafia A, Binyousef FH, Algwaiz A, Almazyed A, Alduaylij T, Alolaiwi O, Alajlan A, Alsuhaibani M, Alenazi KA (2021). Attitudes Towards Complementary and Alternative Medicine Among Pediatricians in Saudi Arabia. Cureus.

[55] Langler A, Boeker R, Kameda G, Seifert G, Edelhauser F, Ostermann T (2013). Attitudes and beliefs of paediatric oncologists regarding complementary and alternative therapies. Complement Ther Med.

[56] Roth M, Lin J, Kim M, Moody K (2009). Pediatric Oncologists' Views Toward the Use of Complementary and Alternative Medicine in Children With Cancer. J Pediatr Hematol Oncol.

[57] Varejao CDS, Santo F (2019). Laser Acupuncture for Relieving Nausea and Vomiting in Pediatric Patients Undergoing Chemotherapy: A Single-Blind Randomized Clinical Trial. J Pediatr Oncol Nurs.

[58] Jones E, Isom S, Kemper KJ, McLean TW (2008). Acupressure for chemotherapy-associated nausea and vomiting in children. J Soc Integr Oncol.

[59] Ghezelbash S, Khosravi M (2017). Acupressure for nausea-vomiting and fatigue management in acute lymphoblastic leukemia children. J Nurs Midwifery Sci.

[60] Evans A, Garretson C, Pedroja E, Malvar J, Margol A, Sposto R, Nelson MB (2016). The use of aromatherapy to reduce chemotherapy-induced nausea in children with cancer; A randomized, double blind, placebo controlled trial. Neuro Oncol.

[61] Wong J, Ghiasuddin A, Kimata C, Patelesio B, Siu A (2013). The Impact of Healing Touch on Pediatric Oncology Patients. Integr Cancer Ther.

[62] Facchini M, Ruini C (2021). The role of music therapy in the treatment of children with cancer: A systematic review of literature. Complement Ther Clin Pract.

[63] Ibrahim HA, Amal AA (2020). The Effectiveness of Play Therapy in Hospitalized Children with Cancer: Systematic Review. J Nurs Pract.

[64] Purrezaian M, Purrezaian H (2022). Group psychodrama for children with leukemia: A brief report. J Psychosoc Oncol.

[65] Mora DC, Kristoffersen AE, Overvåg G, Jong MC, Mentink M, Liu J, Stub T (2022). Safety of Complementary and Alternative Medicine (CAM) treatment among children and young adults who suffer from adverse effects of conventional cancer treatment: A systematic review. Integr Cancer Ther.

[66] Molassiotis A, Fernadez-Ortega P, Pud D, Ozden G, Scott JA, Panteli V, Margulies A, Browall M, Magri M, Selvekerova S (2005). Use of complementary and alternative medicine in cancer patients: a European survey. Ann Oncol.

[67] King N, Balneaves LG, Levin GT, Nguyen T, Nation JG, Card C, Truant T, Carlson LE (2015). Surveys of cancer patients and cancer health care providers regarding complementary therapy use, communication, and information needs. Integr Cancer Ther.

[68] Truant T, Bottorff JL (1999). Decision making related to complementary therapies: a process of regaining control. Patient Educ Couns.

[69] Masten AS (2001). Ordinary magic: Resilience processes in development. Am Psychol.

[70] Delgado-Rodríguez M (2004). Llorca J: Bias. J Epidemiol Community Health.

[71] Malterud K (2011). Kvalitative metoder i medisinsk forskning: en innføring: Universitetsforlaget.

[72] Pritchard-Jones K, Pieters R, Reaman GH, Hjorth L, Downie P, Calaminus G, Naafs-Wilstra MC, Steliarova-Foucher E (2013). Sustaining innovation and improvement in the treatment of childhood cancer: lessons from high-income countries. Lancet Oncol.

